# Immediate Effects of Repetitive Magnetic Stimulation on Single Cortical Pyramidal Neurons

**DOI:** 10.1371/journal.pone.0170528

**Published:** 2017-01-23

**Authors:** Jineta Banerjee, Mary E. Sorrell, Pablo A. Celnik, Galit Pelled

**Affiliations:** 1 The F.M. Kirby Research Center for Functional Brain Imaging, Kennedy Krieger Institute, Baltimore, Maryland, United States of America; 2 The Russell H. Morgan Department of Radiology and Radiological Science, The Johns Hopkins University School of Medicine, Baltimore, Maryland, United States of America; 3 Department of Physical Medicine and Rehabilitation, The Johns Hopkins University School of Medicine, Baltimore, Maryland, United States of America; Chunag-Ang University, REPUBLIC OF KOREA

## Abstract

Repetitive Transcranial Magnetic Stimulation (rTMS) has been successfully used as a non-invasive therapeutic intervention for several neurological disorders in the clinic as well as an investigative tool for basic neuroscience. rTMS has been shown to induce long-term changes in neuronal circuits *in vivo*. Such long-term effects of rTMS have been investigated using behavioral, imaging, electrophysiological, and molecular approaches, but there is limited understanding of the immediate effects of TMS on neurons. We investigated the immediate effects of high frequency (20 Hz) rTMS on the activity of cortical neurons in an effort to understand the underlying cellular mechanisms activated by rTMS. We used whole-cell patch-clamp recordings in acute rat brain slices and calcium imaging of cultured primary neurons to examine changes in neuronal activity and intracellular calcium respectively. Our results indicate that each TMS pulse caused an immediate and transient activation of voltage gated sodium channels (9.6 ± 1.8 nA at -45 mV, p value < 0.01) in neurons. Short 500 ms 20 Hz rTMS stimulation induced action potentials in a subpopulation of neurons, and significantly increased the steady state current of the neurons at near threshold voltages (at -45 mV: before TMS: I = 130 ± 17 pA, during TMS: I = 215 ± 23 pA, p value = 0.001). rTMS stimulation also led to a delayed increase in intracellular calcium (153.88 ± 61.94% increase from baseline). These results show that rTMS has an immediate and cumulative effect on neuronal activity and intracellular calcium levels, and suggest that rTMS may enhance neuronal responses when combined with an additional motor, sensory or cognitive stimulus. Thus, these results could be translated to optimize rTMS protocols for clinical as well as basic science applications.

## Introduction

Transcranial Magnetic Stimulation (TMS) is a non-invasive method of neural activation, currently used as a therapeutic intervention in neurological disorders like depression [1, 2], and migraine [3]; and being investigated for clinical applications in epilepsy [4, 5], movement disorders [6], stroke [7, 8], brain injury [9], and schizophrenia [10, 11]. It is also frequently used as an investigative tool for basic neuroscience [12–17]. Numerous studies with single pulse TMS (sTMS) as well as repetitive TMS (rTMS) have demonstrated that TMS effects can persist for as long as 6 months after the cessation of treatment. Indeed, both clinical [18, 19] and pre-clinical [20–23] studies suggest that TMS can reshape neuronal connections leading to long-lasting changes (neuroplasticity). Nevertheless, the cellular mechanisms underlying these TMS-induced long-term plasticity changes remain poorly understood. This lack of understanding has led to high variability in the responses of patients to TMS treatment in the clinical setting. Therefore, elucidating the cellular mechanisms involved would aid in understanding the neuronal basis of the effect of different rTMS protocols and design better treatment protocols.

Additionally, while the long-term effects of TMS have been investigated using behavioral [24, 25], imaging [26, 27], electrophysiological [20], and molecular approaches [28, 29]; there is limited understanding of the immediate effects of TMS on neurons [30, 31]. Evidence from basic science proposes potential long-term molecular changes associated with rTMS therapy. For example, studies demonstrated that TMS can lead to changes in gene expression, nitric oxide synthase levels, and extracellular dopamine and glutamate levels in the brain [32–34]; TMS has also been shown to increase markers associated with neuronal activity and plasticity such as *c*-*fos* [35, 36] and brain-derived neurotrophic factor (BDNF) [29, 36, 37]. While high frequency stimulation (>5Hz) increases neuronal activity [38–40], low frequency TMS (1–5Hz) has been shown to suppress evoked dendritic calcium activity by activating GABA_B_ receptors [16, 22]. Thus it is evident that most reported studies of TMS address the long-term effects of TMS on neuronal circuits, which can be highly variable depending on the stimulation protocol. A better understanding of the immediate effects of TMS is key to designing better TMS protocols for both clinical as well as basic science investigations.

Here we used patch-clamp electrophysiology and calcium imaging techniques to determine the immediate and short-term responses of cortical neurons to high frequency (20 Hz) rTMS at a single cell resolution. We tested the responses of individual neurons in acute rat neocortical slices as well as cortical neuronal cultures to low intensity 20 Hz rTMS. The results demonstrate that brief rTMS protocols led to immediate activation of specific ion channels thereby leading to an increase in the steady state current of cortical neurons. Longer rTMS protocols led to increases in the calcium responses of the neurons, which in turn could influence molecular and architectural change associated with neuronal plasticity.

## Materials and Methods

All animal procedures were conducted in accordance with the NIH Guide for the Care and Use of Laboratory Animals and approved by the Johns Hopkins University Animal Care and Use Committee.

### Slice preparation and electrophysiology

All whole-cell electrophysiology experiments were done in acute neocortical slices from SD rats of age P10-25, since previous studies have shown that the intrinsic and firing properties of layer IV pyramidal neurons mature to adult-like after the developmental switch at P8 [41]. P10-P25 SD rat pups were anaesthetized using isoflorane and rapidly decapitated. 300 μm coronal brain slices were prepared in ice cold ACSF (composition in mM: NaCl-119, MgSO_4_.7H_2_O-1.2, KCl-2.5, NaH_2_PO_4_-1.15, Glucose-11.0, NaHCO_3_-26.2, CaCl_2_.2H_2_O-2.5, bubbled in 95% O_2_/ 5% CO_2_, pH 7.4, at room temperature (25°C)) and recovered at 34°C. Intracellular recordings were made at room temperature using 5–7 MΩ patch electrodes (internal solution: in mM: Potassium gluconate-127, KCl-10, NaCl-6, Na-phosphocreatine-3.0, MgCl_2_.6H_2_O-1.0, CaCl_2_-1.0, HEPES-20.0, EGTA-10.0, Na-GTP-0.5, Mg-ATP-3.0, Alexa-568- 0.2). Ion channels were pharmacologically blocked by bath application of selective inhibitor of sodium channel conductance tetrodotoxin (Tocris Biosciences), Gamma amino butyric acid (GABA_A_) receptor antagonist SR95531 (Tocris Biosciences), selective N-methyl-D-aspartate (NMDA) receptor antagonist DL-AP5 (Tocris Biosciences), and selective α-amino-3-hydroxy-5-methyl-4-isoxazolepropionic acid (AMPA) and kainate receptor antagonist DNQX (Tocris Biosciences) stock solutions diluted in ACSF. QX314 (Tocris Biosciences) was added to internal recording solution in the patch pipette to block voltage gated sodium channels in the recorded neurons. Whole cell voltage-clamp and current-clamp recordings were obtained from layer 4/5 pyramidal neurons visually identified using DIC optics (Examiner.D1, Carl Zeiss AG). The neurons were filled with fluorescent dye Alexa-568 sodium hydrazide (Life Technologies) for post-hoc confirmation of the morphology. The intracellular current and voltage signals were recorded using a Multiclamp700B amplifier (Axon Instruments) and a Digidata 1440A digitizer (Axon Instruments). The signals were Bessel filtered at 10 KHz. We recorded and analyzed neurons that responded with action potentials (spikes) when depolarized by suprathreshold current injection. Overall 32 neurons were recorded from 14 different SD rats. Pipette access resistance (R_a_) was monitored throughout each experiment. A neuron was excluded from analysis only if R_a_ changed more than 30% during the experiment. Every data point included in the population data is an average of 3 trials of stimulation on a specific neuron.

To test whether TMS stimulation caused electromagnetic interference in the recording setup, control experiments were performed by attaching a Model Cell (Axon Instruments) to the head stage and recording the voltage clamp and current clamp responses to TMS stimulation. When the headstage is connected to the ‘Cell’ mode of the Model Cell, the current exiting the headstage first goes through a circuit with an impedance of 500 MΩ/33 pF (mimicking a cell) and then a resistance of 10 MΩ (mimicking an electrode). Since this electrical circuit is hard-wired and not responsive to TMS stimulation, it was used not only to test the electromagnetic interference due to TMS but also served as a negative control for our whole-cell recording experiments.

### Transcranial magnetic stimulation

A Magstim Rapid2 system (The Magstim Company Ltd.) with a custom-built 25 mm coil (Jali Medical Inc.) was used to deliver stimulation. The coil was positioned approximately 20 mm from the coronal rat brain slice in the recording chamber or the cultured neurons on the imaging platform. The figure-of-eight shaped coil produced a focal field with the maximal intensity at the intersection of the round components. The coil was held at a 45-degree angle so as to target the center of the coil towards the brain slice in the recording chamber and 20 Hz rTMS stimulation was performed at 30% maximal stimulator output. The maximum strength of the magnetic field at the distance of the slice was determined to be 200 mT at 100% maximum stimulator output, as was calculated by mathematical modeling. With 30% power output, the maximum strength of the field at the slice was estimated to be approximately 60 mT.

### Imaging of neuronal co-cultures with mixed glial cells

All calcium imaging experiments were done using cultured primary neurons. Cortical mixed glial cultures were prepared from P0-P2 ICR mouse pups (CD-1®; Harlan Laboratories) and maintained in glial maintenance medium made of Dulbecco’s Modified Eagle’s Medium (DMEM) with 10% defined fetal bovine serum (Hyclone, Logan, UT). Glial cells were allowed to reach confluence, which took approximately 10 days. Then cortical neurons were prepared from E15-E16 ICR mouse embryos (CD-1®; Harlan Laboratories). Neurons were placed on top of a confluent mixed glial bed layer at a density of 0.8–1.0 x10^5 cells per well. Neuron-glia co-cultures were maintained in Neurobasal medium supplemented with B27, 0.5 mM L-glutamine, and allowed to mature for about 8 days prior to the start of the experiments. Cultures were loaded with 1 μM fura-2/AM (Molecular Probes/Invitrogen; 45 min, 37°C) in Hank’s balanced salt solution with 10 mM HEPES buffer (pH 7.2), and then incubated for an additional 30 min at 37°C to allow for complete hydrolysis of the acetoxy-methyl ester group. An inverted Olympus 1X71 (Olympus Corporation) microscope with a dual condenser illumination column was used to image fluorescence intensity changes that are proportional to intracellular calcium concentration during stimulation. A 60 s baseline was recorded, followed by a 10 s, 20 Hz rTMS stimulation. Experiments were carried out for a total of 400 s. Data are reported as percentage change in fura-2 ratio at 340/380 nm excitation relative to values recorded at T = 0 for each neuron. Approximately 30% of the cultured neurons responded to TMS stimulation. Responsive neurons were included in graphed data and used for statistical analysis. Each circle denotes time of peak of a significant calcium concentration change. The same numbers of neurons were observed across all treatment groups. Data analysis was performed on custom MATLAB software.

## Results

### rTMS induces voltage sensitive transient inward currents in cortical neurons

**Fig 1:**
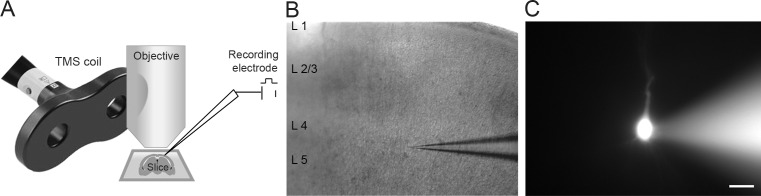
20 Hz rTMS stimulation on single cortical neurons. (A) Diagrammatic representation of the experimental setup highlighting the relative positions of the TMS coil, the acute brain slice, objective and the recording electrode for whole-cell recording experiments. (B) 5X DIC image showing recording pipette in cortical slice to indicate position of recorded neurons in layer 4/5 in cortex. (C) 40X fluorescent image of a recorded neuron filled with Alexa-568. The soma and apical dendrite are visible in the image (Scale bar: 20 μm).

**Fig 2:**
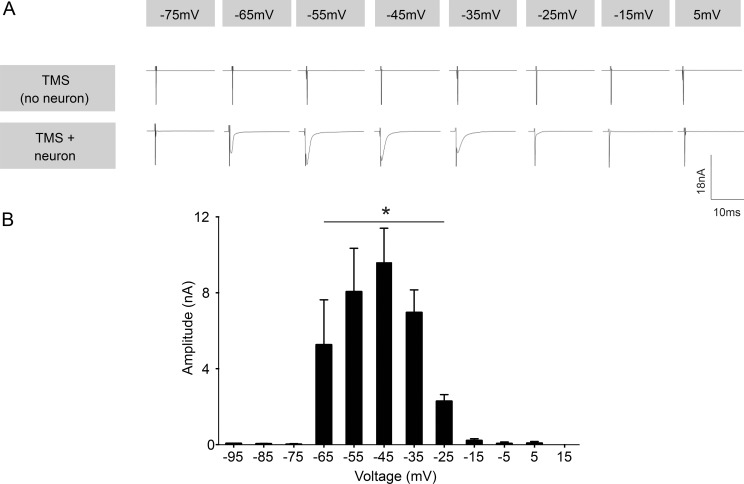
TMS pulses induce transient inward currents at specific voltages. (A) Example voltage clamp traces of TMS induced responses in Model Cell (top panel, no neuron) and cortical neurons (bottom panel, TMS + neuron). (B) Average amplitudes of the TMS-induced current in cortical neurons (n = 10) at different voltages. Error bars represent SEM.

Whole-cell recordings of layer 4/5 pyramidal neurons (somaotosensory cortex) were performed on acute coronal neocortical slices. Fig 1A shows a diagrammatic representation of the experimental setup for rTMS application during whole-cell electrophysiology. Individual neurons were identified visually by their location and morphology (Fig 1B) and filled with a fluorescent dye after recording to ascertain identity (Fig 1C). The recorded neurons had a mean resting membrane potential of -74.03 ± 1.323 mV (corrected for junction potential, n = 13). To investigate the effect of TMS on active neurons, each neuron was held at various membrane voltages (ranging from -95 mV to +15 mV, at 10 mV increments) to simulate different activity levels in the brain. Then 20 Hz rTMS stimulation was applied for a brief period of 500 ms. At voltages ranging from -65 mV to -25 mV, each TMS pulse in the 20 Hz rTMS train caused a significant transient influx of current into the neurons (-65 mV: 5.2 ± 2.3 nA, -55 mV: 8.1 ± 2.3 nA, -45 mV: 9.6 ± 1.8nA, -35 mV: 7.0 ± 1.2 nA, -25 mV: 2.3 ± 0.35 nA, n = 10, Fig 2A and 2B). The transient inward current lasted for several milliseconds with a decay kinetics that fitted a single exponential function with a mean decay time constant (tau) of 3.08 ± 0.84 ms (Fig 2A, TMS + neuron). These transient inward currents were absent in control experiments using a Model Cell (an electronic apparatus mimicking the electrical responses of a neuron) (Fig 2A, top panel, no neuron) thus confirming the biological origin of the observed responses. The control trials with the Model Cell (Fig 2A, top panel, no neuron) further highlighted that the rTMS stimulation did not induce any electrical interference in the headstage amplifier. Thus the results indicate that a single TMS pulse is capable of inducing current influx in the soma of cortical neurons and this current flow is sensitive to the membrane voltage of the cell (Fig 2B). The induction of this current only at specific voltages suggests the involvement of voltage sensitive ion channels.

### rTMS transiently opens voltage gated sodium channels and increases steady state currents

**Fig 3:**
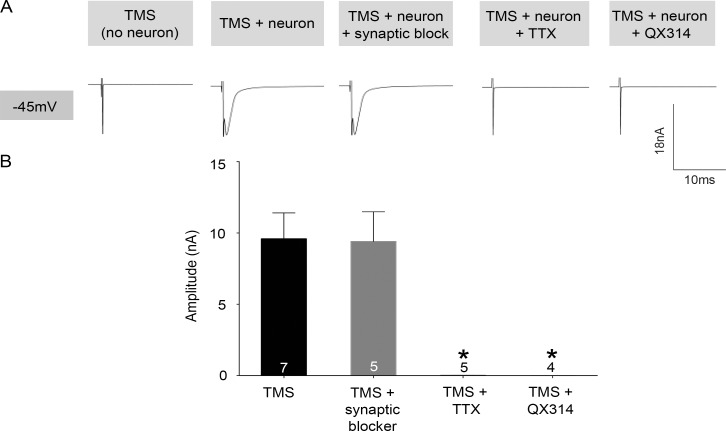
TMS pulses transiently open voltage gated sodium channels. (A) Example voltage clamp traces of TMS induced responses in cortical cells at -45mV in the presence and absence of DNQX, DL-AP5, and SR99531 which are pharmacological agents blocking glutamatergic and GABAergic signaling in the slice. (B) Population data of the mean amplitude of TMS pulse-induced current in cortical neurons during different pharmacological conditions (Numbers within bars indicate sample size for each experimental group). Error bars represent SEM.

To investigate the identity and combinations of ion channels that may be activated by rTMS pulses, different ion channel blockers were added to the recording solution. When all synaptic activity in the brain slice was blocked by simultaneous application of pharmacological agents (DNQX, DL-AP5, and SR95531) in the extracellular ACSF, the 20 Hz rTMS induced currents were not affected (n = 5, Fig 3). This suggests that the primary effect of rTMS on neurons is via activation of ion channels directly on the soma and not due to synaptic activity. Subsequently when voltage gated sodium channels (VGSCs) were blocked in the slice using tetrodotoxin, the rTMS induced current was completely blocked (-45 mV: 12.9 ± 11.02 pA, n = 5, p value = 0.0014, Student’s t test, Fig 3B). Moreover, when the VGSCs were intracellularly blocked specifically in the recorded neuron using QX314 in the recording electrode (-45 mV: -8.53 ± 1.6 pA, n = 4, p value = 0.0036, Student’s t test, Fig 3B), the rTMS induced response was completely eliminated. These results suggest that TMS pulses can cause activation of VGSCs present on the soma of cortical neurons to induce a transient inward current.

**Fig 4:**
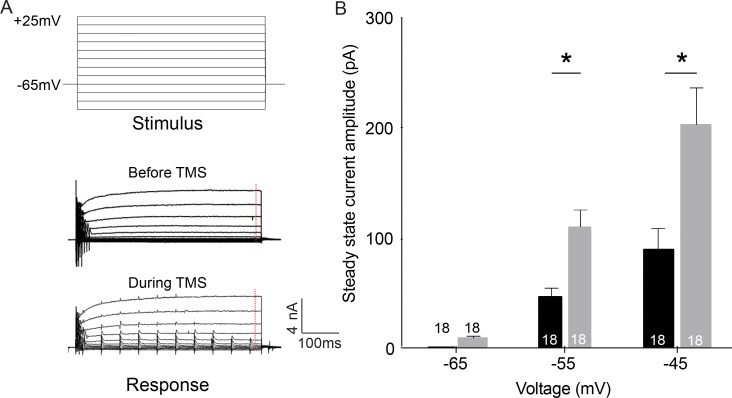
rTMS increases steady state current in cortical neurons. (A) Example voltage clamp traces of (top) stimulus and (bottom) responses (before and during TMS) in cortical neurons (traces have been filtered to remove the TMS artifact). The red dotted line in the response traces denotes the point at which steady state current was measured. (B) Population data of steady state current of cortical neurons at different voltages (Numbers in the bars indicate sample size). Error bars represent SEM.

To investigate the effect of rTMS induced current in the cortical neurons at different states of activation, rTMS stimulation was applied while holding the cells at different depolarized potentials simulating different levels of ongoing network activity (Fig 4A). The changes in steady state currents of the neurons induced by depolarization were measured at the end of the duration of stimulus (Fig 4A: bottom panel, ‘Response’, red dotted line). rTMS pulses caused a significant increase in the steady state current of the neurons at -55 mV and -45 mV, i.e. at voltages close to the action potential threshold (before TMS: -55 mV: 75 ± 8.6 pA, -45 mV: 130 ± 17 pA; after TMS: -55 mV: 116 ± 12 pA, -45 mV: 215 ± 23 pA, n = 18, p value = 0.001, one-way ANOVA, Bonferroni’s multiple comparison test) (Fig 4B). These results suggest that during rTMS stimulation, the close apposition of single TMS pulses lead to repeated activation of VGSCs. The cumulative effect of such repeated sodium influx results in an increase in the steady state current in activated neurons. Such continued elevation of the steady state current by long rTMS protocols can modulate the output of the neurons responding to ongoing cortical activity.

### rTMS induces action potentials and reshapes spike timing

**Fig 5:**
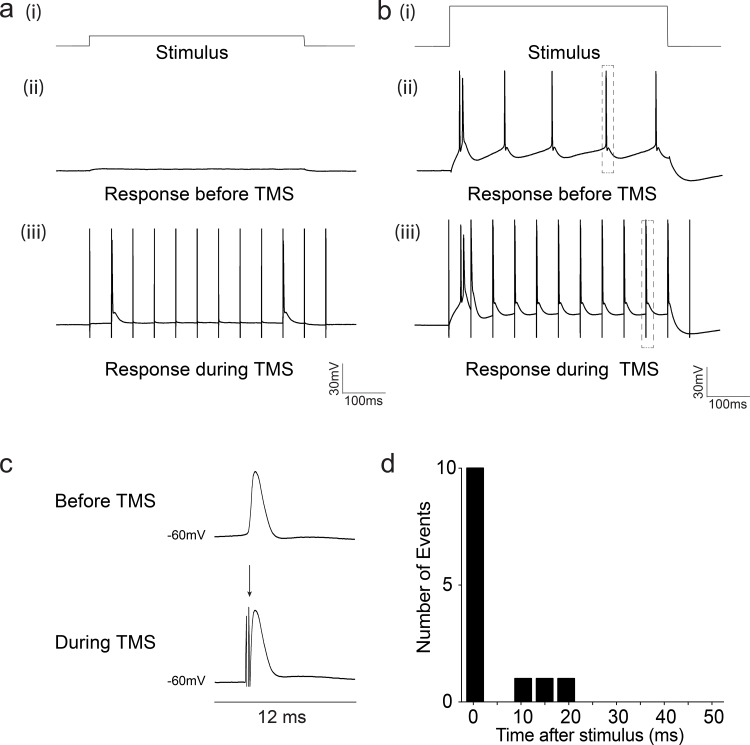
rTMS induces action potentials and reshapes spike timing. (A) Example trace of a neuron showing lower activation threshold during rTMS stimulation (i) 50 pA subthreshold stimulus step (ii) response before rTMS, (iii) response during rTMS (B) Example trace of a neuron showing the effect of rTMS on spike timing (i) 300 pA supratheshold stimulus step (ii) response before rTMS, (iii) response during rTMS. (C) Upper panel: Magnified view of an action potential before TMS (corresponding to grey box in B (ii)), Lower panel: Magnified view of a TMS artifact (arrow head, two pronged artifact) followed by an action potential during stimulation (corresponding to grey box in B (iii)), (D) Representative post stimulus time histogram (PSTH) of the response neuron in (B) during rTMS stimulation.

We examined whether rTMS increases spiking activity of cortical pyramidal neurons during stimulation. Neurons were depolarized using 500 ms current steps in 100 pA increments (Fig 5A(i) and 5B(i)). At supra-threshold stimulation, they responded with trains of action potentials showing characteristic adaptation (Fig 5B(ii)). In 38% of recorded neurons (5 of 13 neurons), 20 Hz rTMS induced action potentials even in presence of subthreshold current stimuli (Fig 5A, number of spikes: before TMS = 0.0 ± 0, during TMS = 1.60 ± 1.2, range of threshold stimulus steps: 0–300 pA). Additionally, in 15% of the recorded neurons (2 of 13 neurons), the timing of neuronal spiking in response to suprathreshold stimulus became correlated to the 20 Hz rTMS stimulation frequency (Fig 5B(i), 5B(ii) and 5B(iii)). Fig 5C shows magnified images of a single action potential (response before TMS), as well as a single two pronged TMS artifact (marked by an arrow) closely followed by an action potential (response during TMS). A post-stimulus histogram (PSTH) (Fig 5D) further confirms that most of the action potential events occurred within 1 ms following the TMS pulse. These results suggest that rTMS is capable of inducing action potentials and reshaping the timing of spikes in response to a stimulus.

### rTMS increases intracellular calcium concentrations

**Fig 6:**
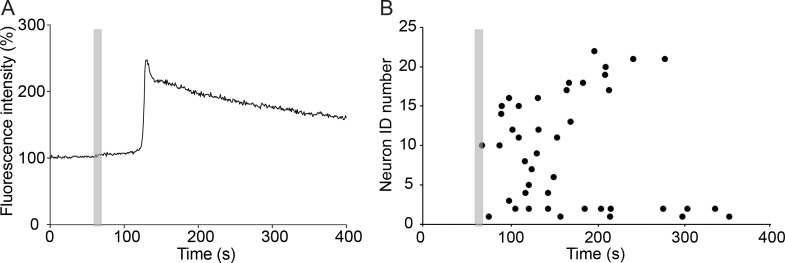
rTMS increases intracellular calcium concentrations in cultured neurons. (A) Example time course plot of the increase in calcium signal in a cultured cortical neuron due to TMS. Grey bar indicates 10 s TMS stimulation. (B) Plot showing peaks of intracellular calcium fluxes (black dots) in individual neurons (n = 23) as they occur after TMS stimulation (grey bar).

Very brief (500 ms) 20 Hz rTMS stimulations were used in our electrophysiology experiments to highlight the immediate changes in neuronal activity due to rTMS. However, in recent literature longer trains of 20 Hz rTMS have been used for various behavioral and electrophysiological experiments. To investigate the effects of a comparable stimulation, 10 s long 20 Hz rTMS was applied to cultured primary neurons and the resulting population neuronal activity was visualized using calcium imaging. For imaging experiments, cortical neurons co-cultured with cortical glia were loaded with the calcium indicator fura-2/AM. After recording baseline levels of intracellular calcium for 60 s, 20 Hz rTMS was delivered for 10 s and the percent change in fura-2 ratio at 340/380 nm excitations was calculated from the images. rTMS induced 153.88 ± 61.94% increases in calcium flux that peaked at 71.25 ± 43.31 s with a following peak at 94.36 ± 50.87 s after stimulation (n = 23) (Fig 6A and 6B). This suggests that rTMS stimulation induces short-term changes in intracellular calcium signaling in cortical neurons.

## Discussion

In the present study we report the immediate effects of TMS on acute brain slices at a single cell resolution using a commercial TMS system and clinically relevant high frequency (20 Hz) rTMS protocols. Our results show that each TMS pulse causes an immediate and transient activation of VGSCs in the soma of cortical neurons. Furthermore, a short duration of rTMS significantly increases the steady state current of the neurons, induces action potentials, and modulates spike timing in these neurons. Longer duration rTMS stimulations lead to delayed increases in intracellular calcium in primary cortical neurons.

A critical consideration for TMS stimulation is that the strength of the magnetic field sensed by a neuron varies according to its distance from the stimulating coil [42, 43]. While the primary motor cortex is closer to the skull, other brain areas such as the amygdala, hippocampus and prefrontal cortex, which are often targeted by TMS, are further away from the coil [44–46]. Given that typical sTMS stimulation in patients is around 70% of maximum (2 T) stimulator output [47], and that the current density magnitude generated in the grey matter is only 10% of maximum current generated by the magnetic pulse [48], neurons located in cortex and subcortical areas are subjected to a magnetic field of approximately 140 mT or lower. Recent studies using mathematical modeling, patch-clamp recordings, and optical imaging showed that a single magnetic pulse of approximately 500 mT was capable of generating an action potential at the soma of neurons located proximal to the coil [22, 31]. In our study, rTMS intensities as low as 60 mT evoked action potentials in a subpopulation of neurons, and increased the steady state currents across all recorded neurons due to the cumulative effect of the pulses delivered in close apposition. Thus, the neuronal mechanisms observed in our experimental setup provide insight to the neuronal activity induced by low intensity rTMS in brain areas that are distal from the coil.

TMS has been hypothesized to affect short-term neuronal activity via several ways: Motor evoked potentials (MEPs) elicited by sTMS pulses in human subjects demonstrate that TMS immediately activates descending neuronal pathways. It has been suggested that TMS may activate cortical neurons antidromically, primarily by eliciting action potentials at axonal bends, bifurcations, or terminations [49–52]. The transient inflow of sodium current and increases in steady state current in the cortical neurons in response to TMS pulses observed in our study are in agreement with previous reports where increases in membrane potential were observed *in vivo* using voltage sensitive dyes [30]. We hypothesize that such TMS induced activation of VGSCs may also facilitate suprathreshold activity in axonal Nodes of Ranvier (where the concentration of VGSCs is high) causing antidromic activity. These mechanisms have been implicated in previous studies as possible ways that TMS affects short-term neuronal activity [49–54].

rTMS also has long-lasting effects that are hypothesized to manifest through long-term potentiation (LTP) and long-term depression (LTD) for high (> 5 Hz) and low frequency (<5 Hz) stimulations, respectively [24, 55]. Recent reports have shown that 20 min long 10 Hz rTMS stimulation protocols can cause changes in postsynaptic receptors and their activity within 3 h post stimulation [20]. Such changes suggest that rTMS leads to recruitment of long-term plasticity mechanisms. While the rTMS induced changes in postsynaptic receptors were dependent on NMDA receptor (NMDAR) activation as expected in long-term plasticity changes, blocking NMDARs during TMS stimulations only partially attenuated the changes [20]. This suggested the involvement of presynaptic factors in rTMS mediated neuroplasticity. Our results show that the immediate current induced by TMS pulses increased the steady state current in the presynaptic compartment in an NMDAR independent manner (Fig 2). Such rTMS induced increase in steady state current in the presynaptic compartment may explain the NMDAR-independent effects seen in the previous reports. Longer duration TMS protocols may cause further increases in steady state current; consequently facilitating NMDAR mediated long-term effects. Furthermore, previous studies have shown that the timing of pre- and post-synaptic action potentials also influence synaptic plasticity. When a post-synaptic spike repeatedly follows a presynaptic spike within a brief time window, LTP occurs, while the reverse order leads to LTD [56–58]. Our study shows that rTMS was capable of influencing the spike timing in a subpopulation of neurons during stimulation. In the clinics, the duration of TMS therapy sessions generally are longer (20 min or more). rTMS induced changes in the spike timing for such long durations of rTMS stimulation may be relevant for spike timing dependent plasticity in neuronal circuits leading to LTP and LTD.

Our study also demonstrated that 10 s of rTMS stimulation leads to changes in intracellular calcium concentrations in the neurons. The significant increase in calcium levels occurred about 70 s after the end of stimulation and lasted for almost 200 ms. The delayed peak suggests that the calcium signal was not caused by rTMS induced action potentials but may be due to the cumulative effect of rTMS induced depolarization of the neuron. The long duration of the calcium signal may be attributed to L-type calcium channels, which are known to undergo slow inactivation [59]. Activation of L-type calcium channels by longer duration rTMS stimulation has also been reported in previous studies [21]. Activation of L-type calcium channels along with NMDAR activation has been implicated in facilitating long-term plasticity changes in neurons [60, 61]. Thus our results suggest that immediate changes in cortical neurons by rTMS may lead to long-term plasticity changes even in brain areas that are exposed to low intensity stimulation.

Although rTMS is used as a therapeutic intervention, only recently we have begun to understand the precise mechanisms by which rTMS affects neurons. Based on results obtained from this study, we propose the following sequence of neuronal events that ultimately lead to short- and long-term changes: Initially, TMS pulses cause transient influx of sodium current into cortical neurons and induce action potentials. Repeated TMS pulses lead to increase in steady state current in neurons that are depolarized due to ongoing circuit activity, and subsequently result in activation of L-type calcium channels or postsynaptic NMDARs or both. This may change postsynaptic receptor recruitment and activity at the synapses leading to long-term plasticity in the cortical circuits. Our results also suggest that rTMS induces significant increases in neuronal activity in the presence of another stimulus. This is supported by the whole-cell electrophysiology findings showing significant increases in steady state current at near threshold and depolarized conditions of excitatory neurons. Our calcium imaging findings further suggest that in the absence of any additional stimulus, rTMS is less likely to cause action potential induced calcium activity. These results together lead us to believe that increased ongoing cortical activity (eg. in the actively engaged brain) augment the effect of rTMS mediated modulation of neuronal activity, and help explain state-dependent variability in neuronal responses [29, 62]. Therefore, our results suggest that therapeutic paradigms incorporating rTMS stimulation with simultaneous motor, sensory, or cognitive tasks could significantly augment the therapeutic potential of rTMS compared to rTMS delivered to a resting brain. Thus, the insights revealed by this study brings us a step closer to understanding the cellular effects induced by TMS and will help us design better TMS protocols to reap maximum benefits of this therapy.
